# Efficacy analysis of the Turnbull–Cutait procedure in refractory perianal Crohn’s disease: a two-centre retrospective study

**DOI:** 10.1007/s00384-026-05148-0

**Published:** 2026-05-14

**Authors:** Qianwen Tu, Huanxin Hu, Mengting Zhang, Kangcheng Luo, Songlin Wan, Daojiang Li, Yi Li, Jia Ke, Zhao Ding

**Affiliations:** 1 Department of Colorectal and Anal Surgery, Inflammatory Bowel Disease Surgical Diagnosis and Treatment Center, Zhongnan Hospital of Wuhan University, Wuhan, 430071 China; 2 Department of General Surgery, The Sixth Affiliated Hospital of Sun Yat-Sen University, Guangzhou, 510655 China

**Keywords:** Crohn’s disease, Turnbull–Cutait pull-through procedure, Refractory perianal disease, Delayed coloanal anastomosis

## Abstract

**Purpose:**

Patients with refractory perianal Crohn’s disease (RpCD) often face rectal resection and permanent ostomy as a final resort. The Turnbull–Cutait (TC) pull-through procedure offers a potential sphincter-preserving alternative, yet evidence supporting its use in RpCD remains scarce. This study evaluated the therapeutic efficacy of the TC procedure in this setting.

**Methods:**

A retrospective analysis was conducted on ten patients with RpCD who planned to undergo TC surgery at Zhongnan Hospital of Wuhan University and the Sixth Affiliated Hospital of Sun Yat-sen University from January 2020 to June 2025; ultimately, eight patients completed the surgery. The median follow-up duration was 14.5 months.

**Results:**

In the per-protocol analysis (*n* = 8), the incidence of postoperative complications during follow-up was 37.5%, including 1 case of anal fistula recurrence, 1 case of anal incontinence, and 1 case of rectovaginal fistula recurrence. Five patients achieved short-term technical success, yielding a short-term technical success rate of 62.5%. Their median gastrointestinal quality of life index (GIQLI) score was 138 points, indicating an overall high quality of life.

**Conclusion:**

The TC procedure may serve as a salvage option to avoid permanent stoma in strictly selected patients with RpCD. Although short-term clinical improvement was observed in some patients, the relatively high complication rate and an uncertain long-term efficacy warrant cautious application and careful patient selection.

**Supplementary Information:**

The online version contains supplementary material available at https://doi.org/10.1007/s00384-026-05148-0.

## Introduction

Crohn’s disease (CD) is a nonspecific, chronic, recurrent inflammatory condition that primarily affects the gastrointestinal tract. Studies have indicated that during a 20-year follow-up period after diagnosis, the cumulative incidence of perianal lesions (across all subtypes) in CD patients can reach up to 42.7%, which represents an independent risk factor for poor long-term prognosis for CD [1]. Treatment of perianal CD requires a multidisciplinary approach combining immunosuppressive therapy, antibiotics, and surgery to adequately control disease progression [2]. When CD is complicated by high rectoanal fistulae, multiple rectovaginal fistulae, or severe rectoanal strictures, combined drug therapy with local incision and drainage or strictureplasty yields poor outcomes [3]. These patients ultimately require proctectomy (or proctocolectomy) and permanent ostomy. To resect the diseased bowel while avoiding a permanent stoma, recent studies have investigated the Turnbull–Cutait (TC) procedure in patients with anorectal CD [4, 5]. The Remzi team at the Cleveland Clinic performed the TC procedure on 15 patients with rectoanal CD and reported lower postoperative complication rates [4]. In a retrospective analysis by Lavryk et al. [5], 4 out of 26 cases of rectovaginal fistula were related to CD, with delayed coloanal anastomosis achieving a 75% treatment success rate. Corte et al. [6] studied 79 patients with rectovaginal fistulas (34 secondary to CD), including 11 who underwent the TC procedure. Compared with other repair techniques, the TC technique achieved a 91% success rate. Patients at risk of ostomy in clinical practice often present with complex conditions (e.g. multiple fistulas and severe stenosis). To explore the applicability of TC surgery in such complex refractory perianal Crohn’s disease (RpCD) patients, we performed a retrospective study. This study aimed to retrospectively evaluate the safety, feasibility, and outcomes of the TC procedure for preserving anal function in this specific, complex RpCD population.

## Materials and methods

### Definition of “refractory”

According to the International Organization for the Study of Inflammatory Bowel Disease (IOIBD) consensus [7], CD is considered “refractory” if it meets at least one of the following four criteria: failure of biologic therapy and advanced small-molecule agents with at least two distinct mechanisms of action; recurrence of CD after two or more intestinal resections; complex perianal disease; and inability to implement appropriate clinical management because of psychiatric or psychological factors.

As this study focuses specifically on perianal Crohn’s disease, only patients who met the criterion of complex perianal disease and had no response to both medical therapy and prior local perianal surgery were included. Patients who met only the other criteria were excluded.

### Definition of outcomes

In this study, the primary endpoint, “short-term technical success”, was operationally defined as a patient status at the last follow-up assessment conducted more than 3 months postoperatively that fulfilled all of the following criteria: the absence of spontaneous or pressure-induced fistula drainage; the absence of perianal abscess; no requirement for further surgical intervention for the primary disease; and successful stoma reversal and preservation of anal function. Conversely, failure to meet any of the above criteria was considered “short-term technical failure.” Permanent stoma or anal incontinence was explicitly regarded as a failure to achieve the core objective of preserving anal function.

### Study design and patient population

This study protocol was approved by the Institutional Review Board. Informed consent was obtained from all patients for the acquisition of patient-related data and materials as well as their use for clinical research. A retrospective analysis of 10 patients with RpCD who were scheduled for the TC procedure between January 2020 and June 2025 at Zhongnan Hospital of Wuhan University and The Sixth Affiliated Hospital of Sun Yat-sen University was performed. Eight patients completed the full TC procedure. The remaining two patients underwent temporary ostomy surgery and reported satisfactory quality of life; thus, they did not proceed with the TC procedure.

### Inclusion and exclusion criteria

This study identified a total of 12 patients who were diagnosed with RpCD and who underwent the TC procedure between January 2020 and June 2025 by reviewing the medical records of Zhongnan Hospital of Wuhan University and the Sixth Affiliated Hospital of Sun Yat‑sen University. The inclusion criteria were a diagnosis of at least one of the following: a rectovaginal fistula, multiple anal fistulas, a rectoperineal fistula, or severe long‑segment anorectal fibrotic stricture; a poor response to both medical therapy and prior local perianal surgical procedures for CD-associated complex perianal conditions, and were facing rectal resection or permanent ostomy as a final resort; a preoperative magnetic resonance imaging or ultrasonography confirming an intact sphincter complex (no full-thickness defect); and patient age falling between 18 and 70 years. The exclusion criteria were emergency surgery (e.g. massive gastrointestinal bleeding, intestinal obstruction, or bowel perforation), preoperative Wexner score > 9, concurrent participation in another clinical study, and the presence of multiorgan dysfunction. On the basis of the above criteria, 10 patients were ultimately included in the study.

Notably, among the two excluded patients, one was excluded because of emergency surgery, and the other was excluded because of participation in another clinical trial. This may have biased our study cohort towards patients with relatively stable conditions eligible for elective surgery, which represents one of the inevitable selection biases in the patient screening process of this retrospective study.

### Patient characteristics and data collection

This study retrospectively collected baseline clinical data from all included patients. The collected variables included demographic information (age, sex, and body mass index), disease-related characteristics (disease duration, type of perianal lesion, and history of previous surgery), preoperative and postoperative imaging examinations (MRI, ultrasound, CT, and colonoscopy), preoperative medication history, preoperative Wexner score, and postoperative outcome (GIQLI).

Preoperatively, all patients underwent colonoscopy to assess the condition of the intestinal mucosa. For patients with mucosal lesions, multi-quadrant biopsies were routinely performed on suspicious areas (such as ulcers, erosions, polypoid hyperplasia, or strictures).

### Basic surgical principles

**Fig. 1:**
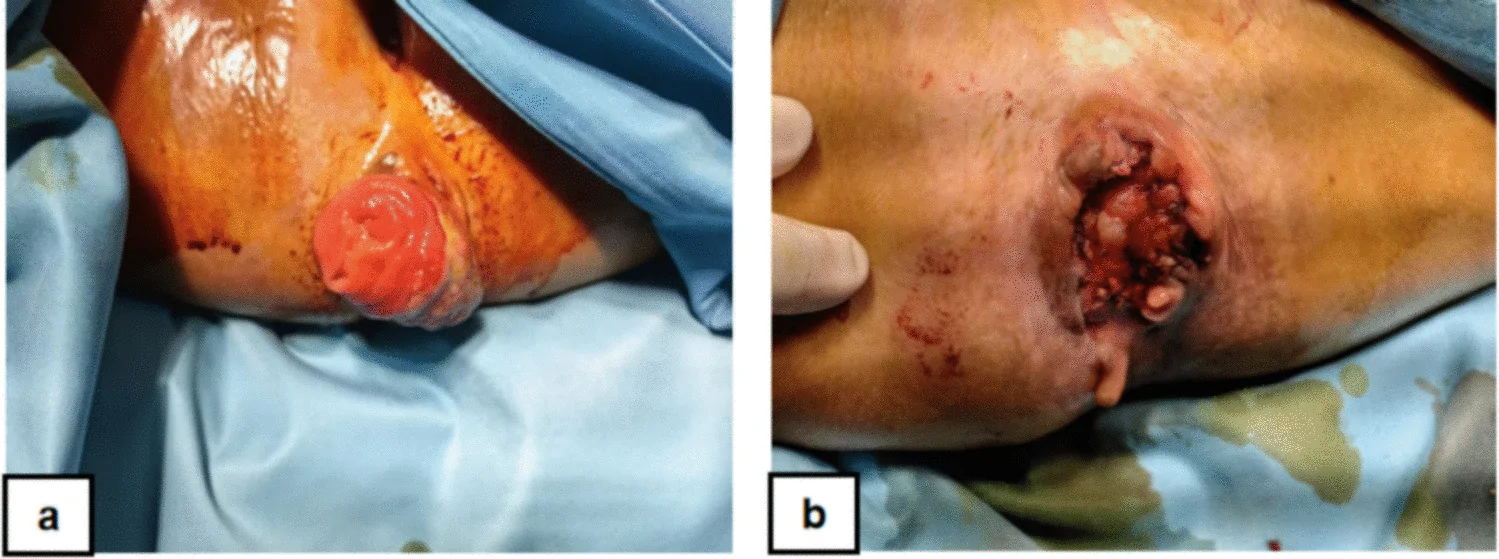
Intraoperative images. **a** Healthy bowel segment pulled through the anus. **b** Anal condition following exteriorized colonic stump resection

All surgeries were performed by experienced colorectal surgical teams from the two centres. The core principles of the TC procedure are as follows: complete resection of diseased perianal and rectal tissue; pull-through of the proximal colon without active inflammation and with good blood supply (Fig. 1a); and delayed coloanal anastomosis to reduce anastomosis-related complications (Fig. 1b).

### Determination of the abdominal approach and proximal transaction line

In all the cases, abdominal procedures—including colonic mobilization, vascular ligation, and proximal colonic transection—were performed laparoscopically. Preoperative colonoscopy and MRI were used to exclude mucosal ulcers or erosions in the intended preserved bowel segment, thereby ensuring the absence of active inflammation. Intraoperative assessment was also integrated, requiring that the serosal surface at the transection site appear normal in colour, without oedema or thickening, and that mesenteric vascular pulsation be satisfactory. In cases of intraoperative uncertainty, a full-thickness wall specimen from the intended transection site was obtained for rapid frozen-section pathology to confirm the absence of active inflammatory cell infiltration in the muscular and submucosal layers. The final transection line was selected at the most distal segment of the healthy bowel that satisfied all the above criteria.

### Specific implementation of the staged surgical strategy

#### Procedure performed at Wuhan University Zhongnan Hospital

The first stage involves creating a colostomy for faecal diversion and managing perianal or perineal abscesses through drainage, combined with medical therapy. The goal is to control infection (clinical symptom improvement, normalization of inflammatory markers, and MRI-confirmed resolution of active inflammation) via faecal diversion and adequate drainage. Phase II: Following infection control, resect the diseased rectum, pull the proximal diseased colon out through the anus, and simultaneously close the colostomy. This stage requires a rigorous assessment of the blood supply and inflammatory status of the pulled-through bowel segment to ensure the safety of anastomosis. Phase III: Resection of the pulled-through segment and completion of the coloanal anastomosis. Anastomotic plane selection is adjusted on the basis of lesion type. For multiple fistula-type lesions, mucosa near the dentate line is resected, and the anastomotic plane is placed below the internal–external sphincter groove to reduce anastomotic recurrence. With respect to long-segment stricture-type lesions, the mucosa near the dentate line may be partially preserved to maintain anal sphincter function.

#### Procedure performed at the Sixth Affiliated Hospital of Sun Yat-sen University

Low-risk type (two-stage approach): In the first stage, the diseased colon and rectum are resected, and healthy bowel is pulled through the anus, with seton drainage of fistulous tracts. This approach is indicated for patients without abdominopelvic or ischioanal abscess and without full-thickness rectal wall inflammation. In the second stage, the exteriorized bowel is resected and anastomosis is completed at the dentate line.

High-risk type (three-stage approach): In the first stage, a diverting ileostomy is created, followed by resection of the diseased bowel and pull-through of healthy bowel, with simultaneous management of fistulas or abscesses. This approach is indicated for patients requiring multivisceral resection, or those with abdominopelvic or ischioanal abscess, or those with full-thickness rectal wall inflammation. In the second stage, the exteriorized colon or rectum is resected and anastomosis is performed at the dentate line. In the third stage, after complete infection control and normalization of inflammatory markers, the ileostomy is closed.

### Statistical analysis

Statistical analysis was performed using SPSS software (version 23.0). Demographic characteristics, disease-related features, and clinical parameters are presented as the mean (standard deviation, SD), median (25th, 75th percentiles), or percentage, as appropriate. For the correlation analysis between continuous variables (e.g. the GIQLI score and disease duration), the nonparametric Spearman’s rank correlation coefficient was used because of the limited sample size. This study is a retrospective cohort study and is reported in accordance with the Strengthening the Reporting of Observational Studies in Epidemiology (STROBE) statement. The completed STROBE checklist is provided in Supplementary Material 1.

## Results

### Patient flow and baseline characteristics

**Fig. 2:**
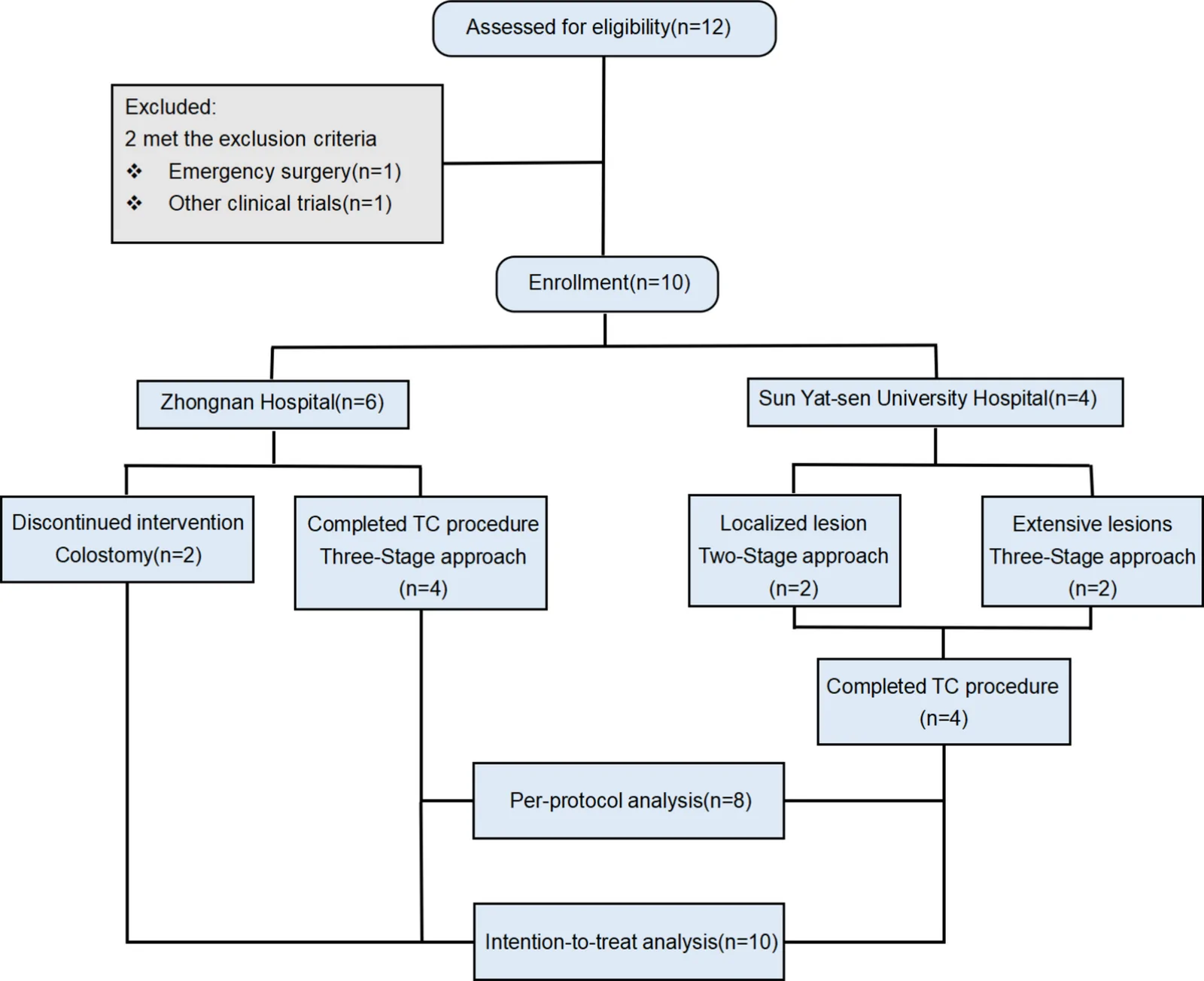
Consolidated standards of reporting trials (CONSORT) flow diagram. Among the 12 initially identified patients, 10 met the inclusion criteria. Two from Zhongnan Hospital discontinued care after stoma creation; the other 4 from the same hospital completed three-stage surgery. At the Sixth Affiliated Hospital, 2 patients underwent two-stage surgery, and 2 underwent three-stage surgery. The outcomes were analyzed separately by intention-to-treat (all 10) and per-protocol (completed TC) approaches

A total of 10 patients with RpCD, who were scheduled for the TC procedure, were included, and their baseline characteristics are presented in Table 1. The patient flow diagram is shown in Fig. 2. Among these patients, 8 completed all planned surgical stages. After undergoing the first-stage temporary ostomy, the remaining 2 patients were satisfied with their quality of life and opted not to proceed with further surgery.

**Table 1:** Baseline characteristics

Parameter	*n*
Age, year, median (IQR)	41(36.5–54)
Preoperative BMI, kg/m^2^, median (IQR)	20.2(18.8–22.9)
Disease duration, year, median (IQR)	7.5(0.9–10.2)
Sex	
Male	5
Female	5
Disease type	
Rectal fistula^b^	2
Complex anal fistula^b^	1
Rectovaginal fistula with concomitant complex anal fistula^b^	2
Complex anal fistula with concomitant anal stenosis^a,b^	2
Rectovaginal fistula with concomitant rectal stenosis^a^	1
Rectovaginal fistula with concomitant anal stenosis^a^	1
Rectovaginal fistula with concomitant rectal and anal stenosis^a^	1

*IQR* interquartile range, *BMI* body mass index

^a^The cases were sourced from Zhongnan Hospital of Wuhan University

^b^The cases were sourced from the Sixth Affiliated Hospital of Sun Yat-sen University

Baseline patient data were available for 10 patients: 5 males and 5 females. Among these patients, 5 had multiple fistulas, including 2 with rectal fistulas, 1 with complex anal fistula, and 2 with both rectovaginal and complex anal fistulas. Five patients had long-segment stricture type, including 2 with both complex anal fistula and anal stenosis, 1 with combined rectovaginal fistula and rectal stenosis, 1 with rectovaginal fistula and anal stenosis, and 1 with rectovaginal fistula, rectal stenosis, and anal stenosis. The median age was 41 (range 36.5–54) years, the median disease duration was 7.5 (range 0.9–10.2) years, the median preoperative body mass index (BMI) was 20.2 (range 18.8–22.9) kg/m^2^, and the median number of prior perianal procedures was 1 (range 0–1.25).

### Preoperative medication management

All 10 patients received medical therapy for CD. Among them, 6 received at least one biologic agent: infliximab (IFX) in 3 patients; IFX followed by adalimumab in 1 patient; IFX followed by ustekinumab in 1 patient; and ustekinumab in 1 patient. The median duration of biologic therapy was 5.5 months (range 2–9). Azathioprine was used in 5 patients. Additionally, 2 patients received thalidomide, one of whom discontinued treatment because of adverse effects of lower limb numbness. Another patient received only hydrocortisone therapy.

### Ending analysis

#### Intent-to-treat analysis (n = 10)

All 10 patients who were scheduled for surgery were included in the analysis. With a median follow-up of 14.5 months, the short-term technical success rate was 50% (5/10). The final statuses were as follows: 5 patients achieved stoma reversal without active disease; 1 patient experienced anal incontinence; 1 patient died due to intraoperatively discovered cancer; and 3 patients remained with a stoma (of these, 2 had a planned temporary stoma but did not undergo subsequent surgery, and 1 did not undergo stoma reversal because of recurrence of the rectovaginal fistula).

#### Per-protocol analysis (n = 8)

Analysis was conducted on the eight patients who completed the full TC procedure. There were no severe perioperative (within 30 days) complications, such as exteriorized bowel necrosis, intra-abdominal bleeding, pelvic infection, or anastomotic leakage. Three patients developed complications requiring intervention, resulting in a complication rate of 37.5% (3/8) (Table 2). According to the time of occurrence and Clavien–Dindo grade [8], the complications were as follows: early complication (≤ 30 days) included one case of anal incontinence (grade IIIb). The patient required daily use of nursing pads, received no specific treatment, and experienced persistent anal incontinence with a GIQLI score of 90. Intermediate complications (31–6 months) included one case of recurrent anal fistula (Grade IIIa), which healed after the incision and drainage. Late complication (> 6 months) included one case of recurrent rectovaginal fistula (Grade IIIb). This patient underwent ileostomy, with a GIQLI score of 130. Additionally, one patient died because of intraoperatively detected cancer progression. The patient had undergone annual colonoscopies prior to surgery. During the most recent preoperative colonoscopy (performed 3 months before surgery), the endoscope could not pass through because of severe anal stenosis; although a biopsy of the stricture was taken, the pathological result was negative. In all previous colonoscopies, since the endoscope was able to pass and no definitive mucosal lesions were observed, routine biopsies were not performed. The short-term technical success rate in this cohort was 62.5% (5/8).

**Table 2:** Postoperative complications

Diagnosis Of complication	*n*	Time of onset	Clavien‒Dindo classification	Treatment	Outcome
Anal fistula	1	5 months after surgery	IIIa	Incision and drainage	Recovered
Rectovaginal fistula	1	1 year after surgery	IIIb	Ileostomy	Ileostomy status
Faecal incontinence	1	1 months after surgery	III-b	None	Faecal incontinence

Complications graded by the Clavien–Dindo system included one case of recurrent anal fistula (Grade IIIa), one case of rectovaginal fistula (Grade IIIb), and one case of faecal incontinence (Grade IIIb)

### Patient-reported quality of life outcomes

**Fig. 3:**
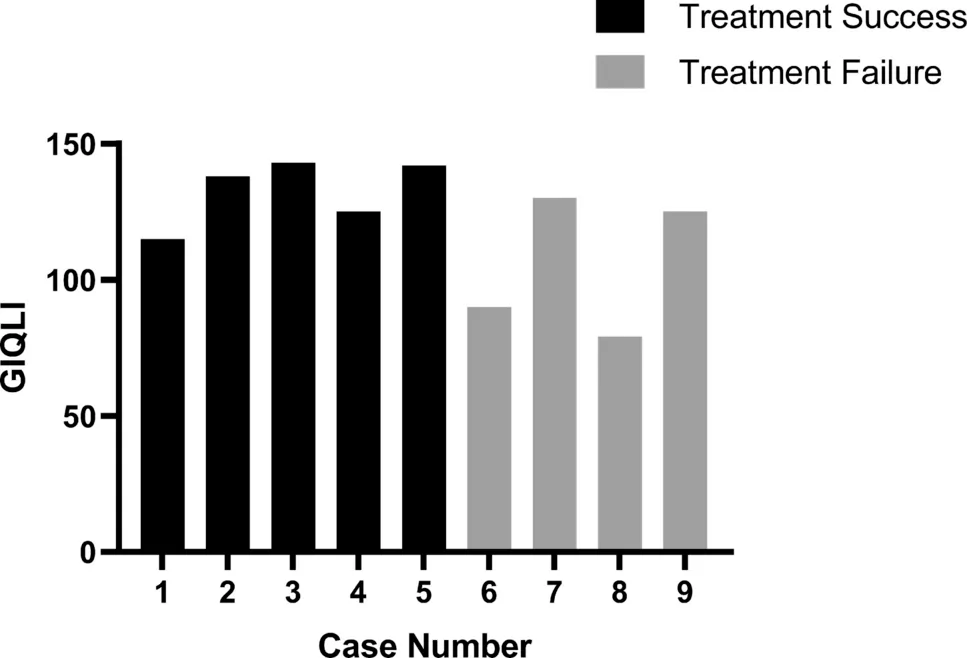
Current GIQLI scores for the 9 patients as assessed in June 2025. The horizontal axis represents the patient number, and the vertical axis represents the GIQLI score. The black bars indicate the treatment-success group, and the grey bars indicate the treatment-failure group

**Fig. 4:**
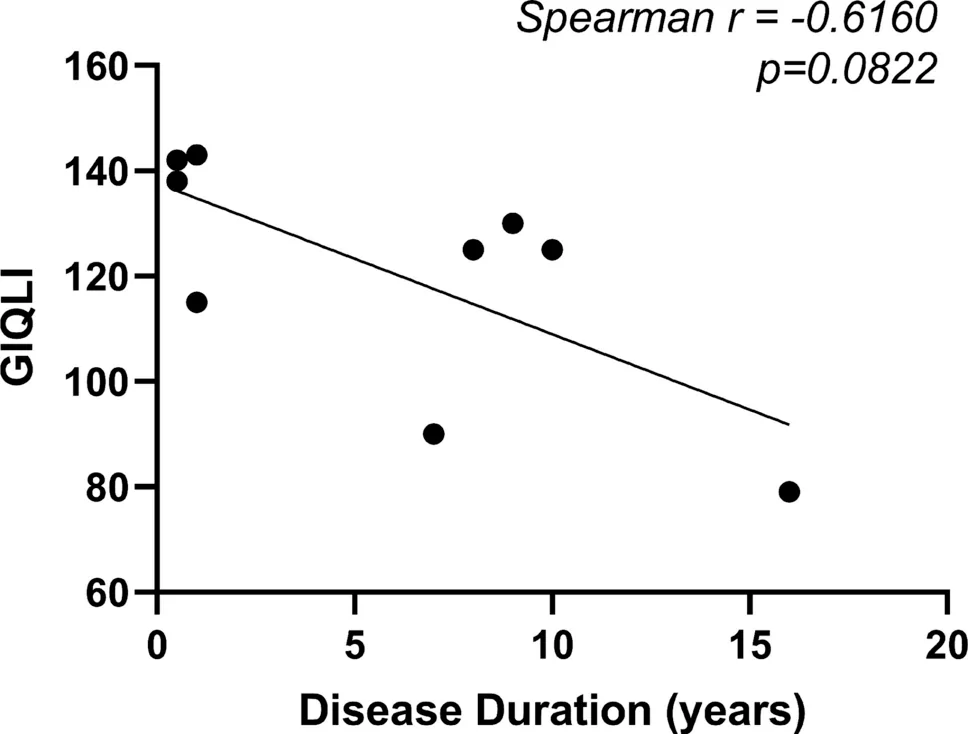
Postoperative quality of life in relation to disease duration. The scatter plot illustrates the distribution of patients’ postoperative GIQLI scores across varying disease durations. Exploratory Spearman correlation analysis revealed a negative association trend, which did not reach statistical significance (rs =  − 0.6160, *p* = 0.082)

As of the follow-up in June 2025, the median GIQLI score among the nine surviving patients was 125.0 (range 90.0–142.0) (Fig. 3). Specifically, the median GIQLI score was 138 (range 120–142) in the five patients with short-term technical success, whereas it was 107.5 (range 81.7–128.7) in the four patients without treatment success. Exploratory analysis based on Spearman’s rank correlation indicated a trend towards a negative association between the postoperative GIQLI scores and disease duration. However, this association did not reach statistical significance in this small sample (rs = −0.6160, 95% CI [−0.90, 0.05],* p* = 0.0822) (Fig. 4).

## Discussion

This two-centre retrospective study investigated the feasibility of the Turnbull–Cutait delayed coloanal anastomosis procedure in avoiding permanent stoma in patients with dual RpCD. Among the eight patients who completed the full surgical protocol, the short-term technical success rate was 62.5% (5/8), and those who achieved success had a good quality of life (median GIQLI score, 138). The complication rate was 37.5% (3/8), with no life-threatening perioperative complications such as exteriorized bowel necrosis or anastomotic leakage. Notably, patients with short-term technical success had a shorter disease duration (mean 2.2 years vs. 9 years) and fewer prior perianal surgeries (mean 0.4 vs. 1). Furthermore, one patient with long-standing RpCD in this cohort was unexpectedly found to have malignancy on postoperative pathology, suggesting that in patients with long-segment stricturing lesions, the TC procedure may have dual diagnostic and therapeutic value.

Consistent with previous studies, TC surgery has often been performed as a last-ditch attempt to preserve anal function before patients undergo rectal resection or permanent ostomy. However, prolonged disease duration, persistent inflammation, and multiple procedures may increase the risk of sphincter injury, leading to excessive scarring, muscle loss, and rectal wall damage, which can have a devastating impact on the quality of life [9]. Among the eight patients who underwent the TC procedure in this study, five achieved short-term technical success, accounting for 62.5% (5/8). In the short-term technical success group, the mean disease duration was 2.2 years, and the mean number of previous perianal surgeries was 0.4. In the short-term technical failure group, the mean disease duration was 9 years, and the mean number of previous perianal surgeries was 1. Compared with patients who experienced short-term technical failure, those who achieved short-term technical success were characterized by a shorter disease duration and fewer previous surgical interventions. These findings suggest that early application of the TC procedure may be more beneficial for patient recovery. One patient in this study had a prolonged disease duration (9 years) and underwent multiple prior procedures (4 procedures, including 3 intestinal repair ostomy procedures and 1 rectal mucosal surgery), with severe intestinal and anal damage. Despite regular medical management post-TC surgery, the rectovaginal fistula recurred. One patient (9-year disease duration) had undergone two prior perianal procedures. Anal fistula recurred after the first perianal surgery and persisted for 5 years without surgical intervention. Chronic, long-term tissue inflammation led to anal muscle dysfunction, making anal incontinence unavoidable after TC surgery. One patient (11-year disease duration) had postoperative pathology indicating malignancy. This case warrants reflection: although the patient underwent annual colonoscopic surveillance, the endoscope could not pass through because of severe anal stenosis before surgery, and the biopsy of the stricture yielded negative results; ultimately, the cancerous change remained undetected. This case highlights the limitations of current screening methods—the presence of a “blind spot” in strictured segments where the endoscope cannot pass and the difficulty of detecting deep mural tumours via mucosal biopsy. Therefore, for patients with RpCD with a long disease duration and complex stricturing lesions, a high index of suspicion for occult malignancy is warranted, even in the presence of negative preoperative biopsies. Studies have demonstrated that CD patients with extensive lesions, a prolonged disease duration, and a younger age at diagnosis exhibit a significantly increased risk of rectal cancer [10]. These findings suggest that earlier surgical intervention may be considered. For high-risk patients with a long disease duration, the TC surgery itself has dual significance—both diagnostic and therapeutic. Owing to sample size limitations, this trend requires validation in larger studies.

Integrated medical and surgical management during the perioperative period is crucial for the long-term prognosis of patients with CD-associated perianal lesions. Compared with surgery alone or anti-TNF therapy alone, combined medical and surgical treatment for CD complicated by anal fistula may yield superior efficacy [11]. IFX effectively induces fistula closure [12], whereas thiopurines combined with metronidazole effectively prevent clinical and endoscopic recurrence after CD surgery [13]. Anal canal function should be monitored and assessed before and after the TC surgery, with local surgical intervention performed when necessary. Concurrently, medications should be administered on the basis of the inflammatory status of the disease to prevent recurrence. In this study, one patient developed perianal abscess and fistula recurrence 4 months postoperatively, potentially related to the discontinuation of IFX treatment due to COVID-19 infection. Symptoms gradually improved after local seton drainage and the resumption of medication. Another patient had a long-standing fistula prior to TC surgery and developed postoperative faecal incontinence, possibly because of the inability to afford effective medications for disease control.

Maggiori et al. [14] demonstrated the feasibility of repeat transanal delayed coloanal anastomosis in patients with failed intestinal anastomosis; among their 24 enrolled patients, one underwent successful secondary surgery for recurrent postoperative pelvic sepsis and achieved favourable long-term function. This presents a potential surgical option for managing such recurrences. However, this study was limited by sample size, follow-up duration, and patient preferences and did not actually attempt a second TC procedure. Our centre suggests that for patients with RpCD who meet the indications for TC surgery, a second TC procedure could be considered a salvage treatment option in cases of recurrence after the first surgery. Nevertheless, the actual efficacy and safety of this approach require future practical attempts and larger-scale studies for evaluation.

In this study, two patients who underwent temporary stoma creation subsequently declined further TC surgery because of their satisfaction with their quality of life, accounting for 20% of the intention-to-treat population. This phenomenon warrants further discussion. From the patient’s perspective, faecal diversion alone can provide significant symptom relief for some patients with complex RpCD, suggesting that a permanent stoma may be an acceptable treatment endpoint for selected individuals. However, from the perspective of disease management, these two patients still face certain risks because of the unresected diseased rectum: the need for lifelong colonoscopic surveillance to monitor for recurrence and an increased risk of malignant transformation due to chronic inflammation in the unresected bowel segment. In contrast, completion of TC surgery not only results in resection of the diseased bowel segment, thereby eliminating the risk of malignancy but also restores intestinal continuity and prevents the formation of a permanent stoma. Therefore, for patients with a strong desire for sphincter preservation and well-preserved sphincter function, the TC procedure may offer superior long-term outcomes. Clinical decision-making should be individualized on the basis of patient preferences, and further validation through large-sample studies is warranted.

This bicentre retrospective study explored the role of the TC procedure in patients with RpCD. In carefully selected patients, the procedure restored intestinal continuity and avoided permanent stoma in a subset of patients, with favourable postoperative quality of life among those with successful outcomes. However, indications remain rare, the procedure is technically demanding, and a notable rate of postoperative complications has been observed; however, the long-term efficacy remains uncertain. Therefore, the TC procedure should currently be considered primarily as a salvage surgical option for carefully evaluated and highly motivated patients wishing to avoid permanent stoma. Exploratory observations from this study suggest that a shorter disease duration and fewer previous perianal surgeries may be associated with better postoperative outcomes, indicating that earlier surgical intervention in selected patients may warrant further investigation. Larger prospective studies with longer follow-up periods are needed to better define optimal patient selection and long-term outcomes.

## Limitations

This study has the following limitations. First, as a retrospective study, it is inevitably subject to selection bias and information bias. Second, the small sample size may have reduced the statistical power, preventing some observed trends (such as the association between disease duration and outcomes) from reaching statistical significance. In addition, the follow-up duration in this study was limited, and the primary endpoint was “short-term technical success”, making it difficult to comprehensively assess long-term complications and recurrence risks associated with the procedure. These findings should therefore be interpreted as short-term outcomes. Longer-term follow-up is warranted to validate functional preservation as well as the risks of recurrence and malignant transformation. Finally, all the cases were sourced from two high-level medical centres within the country, which may limit the generalizability of the findings to broader populations.

## Supplementary Information

Below is the link to the electronic supplementary material.

ESM 1 (DOCX 31.7 KB)

## Data Availability

The deidentified individual patient data that support the findings of this study are available from the corresponding author upon reasonable request.
